# Profiling COVID-19 Vaccine Adverse Events by Statistical and Ontological Analysis of VAERS Case Reports

**DOI:** 10.3389/fphar.2022.870599

**Published:** 2022-06-24

**Authors:** Wenxin Guo, Jessica Deguise, Yujia Tian, Philip Chi-En Huang, Rohit Goru, Qiuyue Yang, Suyuan Peng, Luxia Zhang, Lili Zhao, Jiangan Xie, Yongqun He

**Affiliations:** ^1^ College of Literature, Science and the Arts, University of Michigan, Ann Arbor, MI, United States; ^2^ Department of Cell Biology and Neuroscience, Rutgers University, New Brunswick, NJ, United States; ^3^ Chongqing Engineering Research Center of Medical Electronics and Information Technology, School of Bioinformatics, Chongqing University of Posts and Telecommunications, Chongqing, China; ^4^ National Institute of Health Data Science, Peking University, Beijing, China; ^5^ Department of Medicine, Renal Division, Peking University First Hospital, Peking University Institute of Nephrology, Beijing, China; ^6^ Advanced Institute of Information Technology, Peking University, Hangzhou, China; ^7^ Department of Biostatistics, University of Michigan School of Public Health, Ann Arbor, MI, United States; ^8^ Unit for Laboratory Animal Medicine, University of Michigan Medical School, Ann Arbor, MI, United States; ^9^ Department of Microbiology and Immunology, University of Michigan Medical School, Ann Arbor, MI, United States; ^10^ Center of Computational Medicine and Bioinformatics, University of Michigan Medical School, Ann Arbor, MI, United States

**Keywords:** COVID-19, COVID-19 vaccine, SARS-CoV-2, adverse events, VAERS, ontology, ontology of adverse events, Cov19VaxKB

## Abstract

Since the beginning of the COVID-19 pandemic, vaccines have been developed to mitigate the spread of SARS-CoV-2, the virus that causes COVID-19. These vaccines have been effective in reducing the rate and severity of COVID-19 infection but also have been associated with various adverse events (AEs). In this study, data from the Vaccine Adverse Event Reporting System (VAERS) was queried and analyzed via the Cov19VaxKB vaccine safety statistical analysis tool to identify statistically significant (i.e., enriched) AEs for the three currently FDA-authorized or approved COVID-19 vaccines. An ontology-based classification and literature review were conducted for these enriched AEs. Using VAERS data as of 31 December 2021, 96 AEs were found to be statistically significantly associated with the Pfizer-BioNTech, Moderna, and/or Janssen COVID-19 vaccines. The Janssen COVID-19 vaccine had a higher crude reporting rate of AEs compared to the Moderna and Pfizer COVID-19 vaccines. Females appeared to have a higher case report frequency for top adverse events compared to males. Using the Ontology of Adverse Event (OAE), these 96 adverse events were classified to different categories such as behavioral and neurological AEs, cardiovascular AEs, female reproductive system AEs, and immune system AEs. Further statistical comparison between different ages, doses, and sexes was also performed for three notable AEs: myocarditis, GBS, and thrombosis. The Pfizer vaccine was found to have a closer association with myocarditis than the other two COVID-19 vaccines in VAERS, while the Janssen vaccine was more likely to be associated with thrombosis and GBS AEs. To support standard AE representation and study, we have also modeled and classified the newly identified thrombosis with thrombocytopenia syndrome (TTS) AE and its subclasses in the OAE by incorporating the Brighton Collaboration definition. Notably, severe COVID-19 vaccine AEs (including myocarditis, GBS, and TTS) rarely occur in comparison to the large number of COVID-19 vaccinations administered in the United States, affirming the overall safety of these COVID-19 vaccines.

## Introduction

The emergence of coronavirus disease 2019 (COVID-19), the disease caused by the SARS-CoV-2 virus, has led to the development of vaccines immunizing against SARS-CoV-2. As of 31 December 2021, two COVID-19 vaccines, Moderna’s mRNA-1273 and Johnson and Johnson’s Janssen vaccine Ad26.COV2.S, were authorized for emergency use by the U.S. Food and Drug Administration (FDA), while a third, Pfizer-BioNTech’s Comirnaty, had been fully approved by the FDA for public use. Since their authorization in the United States and other countries, these COVID-19 vaccines have greatly helped to mitigate the spread and severity of COVID-19 around the world.

Although COVID-19 vaccines are generally safe, they have been associated with various adverse events. Three AEs in particular—myocarditis, Guillain-Barré syndrome (GBS), and thrombosis—have been highlighted in the news and by regulatory agencies as potential side effects of one or more of the three FDA-authorized COVID-19 vaccines (https://www.cdc.gov/coronavirus/2019-ncov/vaccines/safety/adverse-events.html). Myocarditis has been reported by the U.S. Centers for Disease Control and Prevention (CDC) as a significant adverse event of the Pfizer-BioNTech and Moderna COVID-19 vaccines, especially after the second dose (https://www.cdc.gov/coronavirus/2019-ncov/vaccines/safety/myocarditis.html). In April 2021, the CDC and FDA temporarily halted the Johnson & Johnson COVID-19 vaccine administration after six U.S. cases of cerebral venous sinus thrombosis (CVST) with thrombocytopenia were reported (https://www.cdc.gov/media/releases/2021/s0413-JJ-vaccine.html). In July 2021, the FDA attached a warning that GBS, an autoimmune and post-infectious immune disease, could be a side effect of the Johnson & Johnson COVID-19 vaccine (https://www.fda.gov/news-events/press-announcements/coronavirus-covid-19-update-july-13-2021).

In the United States, the Vaccine Adverse Event Reporting System (VAERS) is a web-based vaccine adverse event self-reporting system developed by the CDC and FDA (https://vaers.hhs.gov/) (Chen et al., 1994; Singleton et al., 1999). Historically, VAERS has been used to collect and report various vaccine adverse events (AEs), which include medical conditions that occur after vaccination but may not necessarily be caused by the vaccination. Case report data from VAERS is available for download by the public and can be analyzed using various methods. The VAERS AE data were standardized using the Medical Dictionary for Regulatory Activities (MedDRA), a standard terminology for recording and reporting adverse vaccine or drug event data (Brown, 2003). However, MedDRA has several shortcomings including the lack of textual definitions and a well-defined hierarchical structure (Sarntivijai et al., 2012; He, 2014; He, 2016). As a result, the MedDRA hierarchy is usually not used for AE classification. To better support adverse event representation and classification, the community-based Ontology of Adverse Events (OAE) has been developed (He et al., 2014). Enriched (i.e., statistically significant) MedDRA terms from VAERS data analysis can be mapped to the OAE terms, which can be further analyzed using the more accurate OAE AE classification. Multiple studies have shown that the method of the MedDRA-OAE term mapping followed by OAE-based AE classification enables valuable AE classification and data analysis (Sarntivijai et al., 2012; Xie et al., 2016a; Xie et al., 2016b; Wang et al., 2017; Xie et al., 2018; Ngai et al., 2022).

Recently, we developed the COVID-19 Vaccine Knowledge Base (Cov19VaxKB) (http://www.violinet.org/cov19vaxkb/), which contains a comprehensive list of COVID-19 vaccines that are undergoing development or have been authorized for public use, as well as links to relevant publications, news articles, and a VAERS case report analysis tool (Huang et al., 2021). The VAERS case report analysis tool employs three statistical measures to determine significantly enriched AEs for a specific vaccine: case report frequency, proportional reporting ratio (PRR), and Chi-square statistic. This statistical method has also been used in previous studies analyzing VAERS case reports, such as for influenza vaccines (Sarntivijai et al., 2012).

This study used Cov19VaxKB’s VAERS statistical analysis tool and OAE to analyze VAERS case reports associated with FDA-authorized COVID-19 vaccines, particularly the Pfizer-BioNTech (i.e., Pfizer), Moderna, and Johnson & Johnson (i.e., Janssen) vaccines. We generated a list of enriched AEs, which were further analyzed with an ontology-based classification and literature search. Also, three AEs of concern—thrombosis, GBS, and myocarditis—were further analyzed for statistical significance according to sex, age, and dose number.

## Methods

### VAERS Data Collection and Cov19VaxKB Data Processing

Case report data from the VAERS database was downloaded on 7 January 2022, which included all VAERS data processed as of 31 December 2021. The data were then uploaded to the VIOLIN Cov19VaxKB server (Huang et al., 2021). A server-side script was used to parse and filter case report data for COVID-19 vaccines, which include the Pfizer, Moderna, and Janssen vaccines. This data was then formatted to distinguish specific features such as VAERS report year, United States state or territory, age and sex of vaccine recipient, and vaccine name.

### VAERS Statistical Data Analysis

To analyze VAERS data for each of the three FDA-authorized or approved COVID-19 vaccines (Pfizer, Moderna, and Janssen) in comparison to all vaccines in VAERS, four statistical measures were calculated: Pearson’s Chi-square statistic without Yates’s correction, Proportional Reporting Ratio (PRR) (Evans et al., 2001), case report frequency, and crude reporting rate. Case report frequency is defined as the number of AE case reports for that specific AE divided by the total number of AE case reports for the vaccine (Sarntivijai et al., 2012). Crude reporting rate is defined as the number of AE case reports for a specific AE divided by the total number of vaccine doses administered for the vaccine (Suragh et al., 2018).

Cov19VaxKB’s VAERS case report analysis tool utilizes three statistical criteria were then used to determine if an AE is significantly enriched for a specific COVID-19 vaccine: 1) the total number of case reports for the vaccine is at least 3 and case report frequency >0.2%, 2) PRR >2, and 3) Chi-square statistic >4 (Sarntivijai et al., 2012). Based on these criteria, we obtained a list of significantly enriched AEs for the Janssen, Moderna, and Pfizer vaccines. In addition, sex-, age-, and dose-specific adverse event data of these three vaccines were searched and extracted separately from the CDC WONDER’s VAERS database for three AEs: myocarditis, GBS, and thrombosis. Using this data, we then calculated and compared the PRR and Chi-square statistics for different sexes (male and female), age groups (ranging from 18 to 64), and dose number (doses 1–3 doses for Pfizer and Moderna vaccines, and 1 dose for Janssen vaccine). Only dose 1 of the Janssen vaccine was included in this analysis since insufficient data was available for doses 2 and 3 of the Janssen vaccine.

### Ontology-Based AE Classification and Modeling

The list of enriched AEs, which were coded using MedDRA, was mapped to the Ontology of Adverse Events (OAE) (He et al., 2014) and then classified using the OAE-based classification method. Specifically, the Ontofox tool was used to extract a list of enriched AEs and other terms associated with the list terms (Xiang et al., 2010). The Protege-OWL editor (Rubin et al., 2007) was then used to display the terms of the Ontofox output. For ontology modeling, OAE was also used to define the thrombosis with thrombocytopenia syndrome (TTS) adverse event and its five level subtypes based on Brighton Collaboration’s case definitions (Wise et al., 2007). Furthermore, related OAE terms were imported to the Coronavirus Infectious Disease Ontology (CIDO) (He et al., 2020; Huffman et al., 2021), which was further used to model the relations between COVID-19 vaccines and the confirmed AEs associated with these vaccines.

### Literature Mining of Enriched Adverse Events

We identified literature papers related to the enriched AEs for the Pfizer, Moderna, and Janssen vaccines on PubMed and Google Scholar. These articles were then annotated and abstracted in a Microsoft Excel table (Supplementary File S1).

## Results

### General VAERS Data Collection and Analysis *via* Cov19VaxKB

As of 31 December 2021, the VAERS database included a total of 717,577 AE case reports for all FDA-authorized or approved COVID-19 vaccines, including 323,185 reports for the Pfizer COVID-19 vaccine, 329,056 for the Moderna COVID-19 vaccine, 63,741 for the Janssen COVID-19 vaccine, and 1,595 for COVID-19 vaccines for which the manufacturer is unknown. Among the Pfizer, Moderna, and Janssen vaccines combined, the ten most frequently reported adverse events were headache (17.5% case report frequency), pyrexia (14.8%), fatigue (14.6%), chills (12.7%), pain (12.6%), dizziness (10.0%), nausea (9.9%), pain in extremity (9.5%), myalgia (6.0%), and arthralgia (5.9%). According to data from the CDC, 296,870,831 Pfizer vaccines were administered in the United States as of 31 December 2021, while 194,260,394 Moderna vaccines and 17,640,334 Janssen vaccines were administered in the US during that same time period (https://covid.cdc.gov/covid-data-tracker/#vaccinations_vacc-total-admin-rate-total). As of 31 December 2021, the crude reporting rate for all adverse events was 0.361% for the Janssen vaccine, 0.169% for the Moderna vaccine, and 0.109% for the Pfizer vaccine (Table 1).

**TABLE 1:** Total VAERS AE case reports, total vaccine doses administered in the US, and crude reporting rates for Janssen, Moderna, and Pfizer vaccines as of 31 December 2021.

	Janssen Vaccine	Moderna Vaccine	Pfizer Vaccine
Total # of AE case reports	63,741	329,056	323,185
Total # of vaccines administered	17,640,334	194,260,394	296,870,831
Crude reporting rate	0.361%	0.169%	0.109%

Our VAERS data analysis found that females had a higher number of COVID-19 vaccine AE case reports compared to males. Overall, the total COVID-19 vaccine AE case number for females (481,574) was approximately 2.3-fold the case number for males (209,794) by the end of 2021. This trend can also be observed when comparing the top 10 AEs for both biological sexes (Table 2). Specifically, for each of the top 10 AEs, the total number of cases in females was between 2.6- and 3.84-fold the case number in males. Compared to the males, the females also had higher frequencies for each of the top 10 AEs among total case reports for the corresponding biological sex. For example, for the nausea AE, there were 14,415 (6.9% of 209,794) male case reports and 55,307 (11.5% of 481,574) female case reports (Table 2). The female-to-male case report ratio for the nausea AE is 1.667. The most common AE, headache, was reported in 19.6% of all female case reports and 13.5% of all male case reports. Overall, the female-to-male case report ratios for the top 10 AEs ranged from 1.14 to 1.667 (Table 2).

**TABLE 2:** Profiles of COVID-19 vaccine-associated Top 10 AEs in females and males. Data is derived from VAERS case reports from all COVID-19 vaccines as of 31 December 2021.

#	AE	# Of cases in females	% in total female cases	# Of cases in males	% in total male cases	F/M case ratio	F/M % ratio
1	Headache	94,367	0.196	28,300	0.135	3.33	1.452
2	Pyrexia	74,910	0.156	28,802	0.137	2.6	1.139
3	Fatigue	77,049	0.16	25,447	0.121	3.03	1.322
4	Chills	66,146	0.137	23,066	0.11	2.87	1.245
5	Pain	67,849	0.141	20,731	0.099	3.27	1.424
6	Dizziness	51,097	0.106	19,553	0.093	2.61	1.14
7	Nausea	55,307	0.115	14,415	0.069	3.84	1.667
8	Pain in extremity	51,915	0.108	14,852	0.071	3.5	1.521
9	Myalgia	30,550	0.063	11,708	0.056	2.61	1.125
10	Arthralgia	30,126	0.063	11,350	0.054	2.65	1.167

**FIGURE 1:**
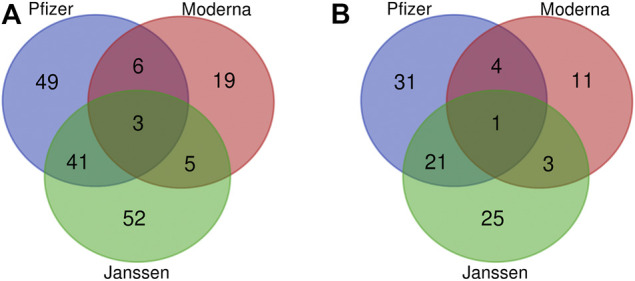
Venn diagram of significant AEs associated with the Janssen, Moderna, and Pfizer vaccines. **(A)** Non-trimmed version of 175 AE terms. **(B)** Trimmed data of 96 AE terms after removing non-medically relevant AEs. The criteria for significance selection included Chi-square statistic >4, PRR >2, and case report frequency >0.2%.

Using the criteria specified in the Methods section, we identified a total of 175 statistically significant enriched adverse events among all COVID-19 vaccines in VAERS (Figure 1A). These enriched AEs are listed in Supplementary File S2. However, several of these enriched AEs were not necessarily medical conditions, including tests or interventions such as “anticoagulant therapy” and “blood test” or administration errors such as “product administered to patient of inappropriate age.” We chose to focus on AEs related to medical conditions. After trimming out non-medically relevant AEs, the total number of enriched AEs was 96. The number of enriched AEs for each vaccine was 57 for the Pfizer vaccine, 20 for the Moderna vaccine, and 51 for the Janssen vaccine (Figure 1B). Of the 96 enriched AEs, 31 were enriched only in the Pfizer vaccine, 11 only in the Moderna vaccine, and 25 only in the Janssen vaccine. Twenty-one AEs were enriched only in the Pfizer and Janssen vaccines, 4 AEs were only enriched in the Pfizer and Moderna vaccines (atrial fibrillation, pharyngeal swelling, vaccination site pain, and Bell’s palsy), and 3 AEs were only enriched in the Moderna and Janssen vaccines (fatigue, chills, and impaired work ability). One adverse event was shared among all three vaccines: ageusia (i.e., loss of taste). Of the three COVID-19 vaccines in our analysis, Moderna was associated with the least number of enriched AEs.

### Hierarchical Classification of Adverse Events Based on the OAE

**FIGURE 2:**
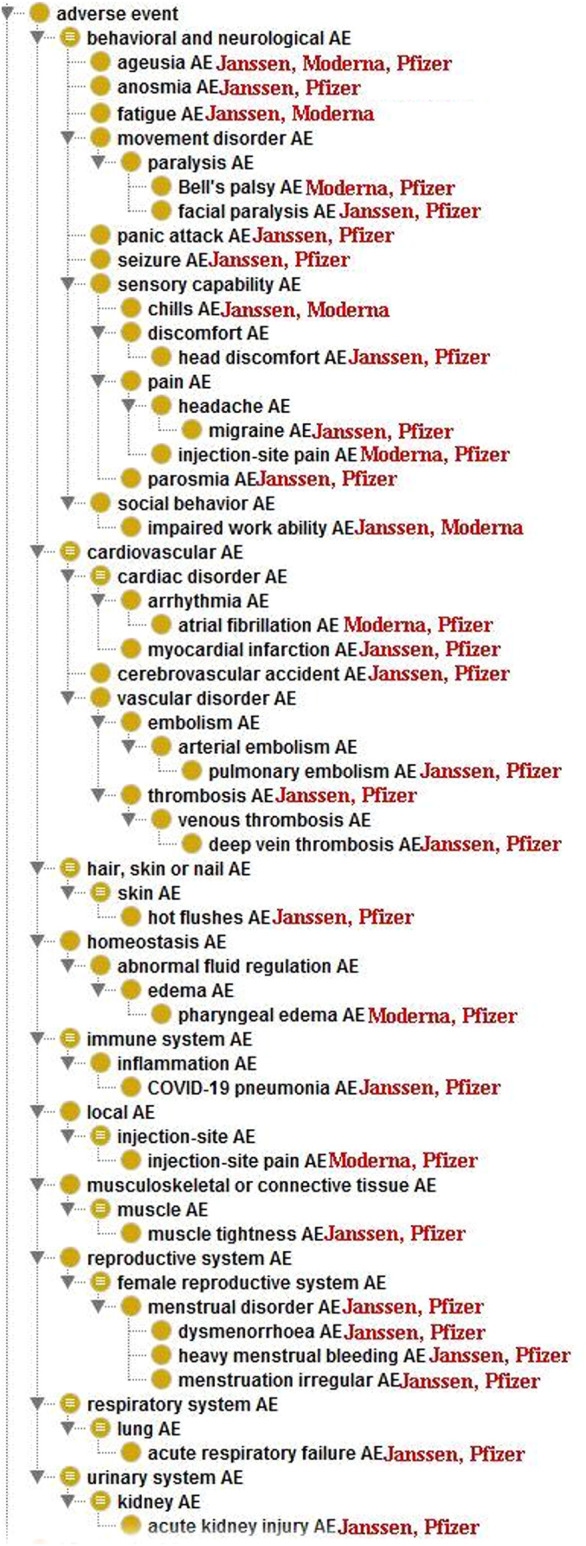
Ontological classification of statistically significant AEs associated with at least 2 of the three COVID-19 vaccines using the Ontology of Adverse Events (OAE).

To further study the 96 statistically significant AEs associated with the Janssen, Moderna, and Pfizer COVID-19 vaccines, we classified these AEs using the OAE-based classification method (Figure 2). Such AE classification provides us a more comprehensive hierarchical visualization of the identified AEs and allows us to conveniently identify relationships between AEs that are significantly enriched for COVID-19 vaccines. Table 3 summarizes the enriched AE results based on the OAE classification.

**TABLE 3:** Statistically significant medically relevant AEs associated with the Janssen, Moderna, and Pfizer COVID-19 vaccines.

Adverse Event	Vaccine	Count	PRR	*x* ^2^
Behavioral and neurological AE (38) Janssen (22) Moderna (6) Pfizer (23)				
Ageusia	Janssen	439	2.13	241.48
	Moderna	1866	2.08	654.59
	Pfizer	2,381	3.26	1944.30
Anosmia	Janssen	344	2.18	199.33
	Pfizer	2007	3.92	2070.10
Anxiety	Pfizer	4,279	2.21	1763.70
Cold sweat	Janssen	659	2.50	535.31
Dysgeusia	Pfizer	3,391	2.64	1985.12
Fatigue	Janssen	10,913	2.01	5,538.40
	Moderna	47,860	2.00	16,620.00
Impaired work ability	Janssen	656	2.20	393.88
	Moderna	2,714	2.09	964.26
Nervousness	Pfizer	1,212	2.05	411.43
Night sweats	Janssen	314	2.04	151.81
Panic attack	Janssen	139	2.13	76.22
	Pfizer	672	2.64	391.61
Bell’s palsy	Moderna	1,384	2.21	558.43
	Pfizer	1728	3.36	1,467.30
Facial paralysis	Janssen	274	2.10	144.77
	Pfizer	1,333	2.61	761.59
Seizure	Janssen	650	2.50	527.36
	Pfizer	2,373	2.10	860.96
Chills	Janssen	11,387	2.31	8,299.80
	Moderna	12,512	19.19	15,573.00
Chest discomfort	Pfizer	8,180	2.59	4,673.10
Head discomfort	Janssen	362	2.58	315.13
	Pfizer	1,531	2.83	1,008.7
Limb discomfort	Moderna	2,380	2.17	929.03
Oropharyngeal discomfort	Pfizer	781	3.41	675.46
Throat irritation	Pfizer	3,323	2.57	1854.5
Disorientation	Janssen	308	2.08	157.98
Vertigo	Pfizer	3,859	2.13	1,450.80
Feeling abnormal	Janssen	2,474	2.11	1,340.3
Feeling of body temperature change	Janssen	217	2.87	234.21
Hypoesthesia oral	Pfizer	2,604	2.79	1,679.20
Axillary pain	Pfizer	3,002	2.68	1802.00
Breast pain	Pfizer	1,017	2.95	716.01
Chest pain	Pfizer	11,968	2.52	6,524.10
Eye pain	Janssen	425	2.11	227.26
Headache	Janssen	15,890	2.24	11,188.00
Migraine	Janssen	1,239	2.71	1,198.10
	Pfizer	4,132	2.04	1,396.60
Lymph node pain	Pfizer	2,702	3.02	1977.50
Oropharyngeal pain	Pfizer	6,060	2.07	2,141.30
Parosmia	Janssen	138	2.25	87.37
	Pfizer	704	3.19	556.56
Sensory disturbance	Janssen	224	2.60	196.94
Throat tightness	Pfizer	3,708	2.28	1,625.5
Unconsciousness	Janssen	1831	2.26	1,177.80
Unresponsive to stimuli	Janssen	632	2.02	299.13
Sleep disorder	Janssen	1,061	2.23	659.92
Cardiovascular AE (14) Janssen (9) Moderna (1) Pfizer (10)				
Hypertension	Pfizer	3,327	2.49	1754.30
Atrial fibrillation	Moderna	1,145	2.11	415.38
	Pfizer	1,251	2.50	660.61
Decreased heart rate	Janssen	144	2.02	67.81
Heart rate irregular	Pfizer	943	2.63	548.00
Increased heart rate	Pfizer	6,946	2.27	3,050.10
Palpitations	Pfizer	7,385	2.67	4,427.80
Myocardial infarction	Janssen	162	2.11	84.57
	Pfizer	740	2.35	345.59
Cerebrovascular accident	Janssen	439	2.94	498.29
	Pfizer	1,391	2.11	511.72
Epistaxis	Janssen	309	2.17	178.72
Contusion	Janssen	644	2.03	311.33
Pulmonary embolism	Janssen	584	4.57	1,351.53
	Pfizer	1,339	2.30	599.16
Thrombosis	Janssen	1,004	6.66	3,716.05
	Pfizer	1737	2.38	830.80
Pulmonary thrombosis	Janssen	139	5.76	433.54
Deep vein thrombosis	Janssen	524	5.85	1,667.82
	Pfizer	943	2.15	361.52
Ear AE (4) Pfizer (4)				
Deafness	Pfizer	809	2.03	269.64
Ear discomfort	Pfizer	1,362	3.00	982.30
Hypoacusis	Pfizer	781	2.50	411.39
Tinnitus	Pfizer	6,933	2.92	4,844.67
Eye AE (2) Janssen (2)				
Vision blurred	Janssen	1,136	2.81	1,178.67
Visual impairment	Janssen	519	2.26	332.92
Female reproductive system AE (5) Janssen (4) Pfizer (5)				
Menstrual disorder	Janssen	231	2.10	121.37
	Pfizer	1,436	4.18	1,592.20
Dysmenorrhoea	Janssen	173	2.08	89.31
	Pfizer	1,090	4.25	1,230.30
Heavy menstrual bleeding	Janssen	444	2.49	355.35
	Pfizer	2,508	4.77	3,183.13
Menstruation irregular	Janssen	316	2.17	182.57
	Pfizer	1875	4.05	2009.78
Vaginal hemorrhage	Pfizer	870	2.95	612.07
Investigation result abnormal AE (1) Pfizer (1)				
Oxygen saturation decreased	Pfizer	942	2.10	340.84
Homeostasis AE (5) Janssen (2) Moderna (2) Pfizer (2)				
Edema limbs	Moderna	10,143	2.26	4,365.30
Pharyngeal edema	Moderna	1810	2.20	722.26
	Pfizer	2,313	3.49	2071.88
Hongue edema	Pfizer	2,421	2.38	1,159.44
Thirst	Janssen	200	2.33	136.87
Hypoxia	Janssen	291	2.12	157.46
Immune system AE (4) Janssen (1) Pfizer (4)				
COVID-19 pneumonia	Janssen	385	3.75	664.63
	Pfizer	1,412	4.06	1,517.44
Myocarditis	Pfizer	1,542	4.09	1,669.49
Pericarditis	Pfizer	975	2.93	676.20
Lymphadenopathy	Pfizer	11,810	2.29	5,303.36
Injury AE (1) Janssen (1)				
Head injury	Janssen	310	2.07	156.62
Local AE (9) Moderna (9) Pfizer (1)				
Injection-site erythema	Moderna	5,670	11.31	12,500.15
Injection-site induration	Moderna	1,205	10.47	2,557.19
Injection-site mass	Moderna	838	5.08	1,109.98
Injection-site pain	Moderna	9,006	4.08	9,701.66
	Pfizer	6,436	2.23	2,708.10
Injection-site pruritus	Moderna	20,473	488	26,765.79
Injection-site rash	Moderna	11,805	3.71	11,407.69
Injection-site reaction	Moderna	1,160	8.68	2,240.94
Injection-site swelling	Moderna	4,312	5.54	6,180.23
Injection-site warmth	Moderna	3,011	8.62	5,805.98
Musculoskeletal or connective tissue AE (2) Janssen (2) Pfizer (1)				
Muscle spasm	Janssen	1,298	2.62	1,168.26
Muscle tightness	Janssen	272	2.27	176.34
	Pfizer	1,050	2.00	337.76
Nervous system AE (1) Pfizer (1)				
Paresthesia oral	Pfizer	3,627	2.94	2,550.65
Respiratory system AE (4) Janssen (3) Pfizer (2)				
Acute respiratory failure	Janssen	204	3.11	255.31
	Pfizer	840	3.68	801.76
Dyspnoea exertional	Janssen	173	2.87	186.62
Sinus congestion	Janssen	133	2.11	70.86
Respiratory tract congestion	Pfizer	1916	2.33	879.76
***Serious adverse event*** (1) Janssen (1)				
death	Janssen	919	2.02	439.26
Skin AE (4) Janssen (2) Moderna (1) Pfizer (2)				
Flushing	Pfizer	4,879	2.32	2,225.60
Hot flushes	Janssen	462	2.38	335.43
	Pfizer	1792	2.16	694.20
Hyperhidrosis	Janssen	3,612	2.71	3,554.09
Rash pruritic	Moderna	5,336	2.08	1877.20
Urinary system AE (1) Janssen (1) Pfizer (1)				
Acute kidney injury	Janssen	137	2.36	97.44
	Pfizer	744	3.95	772.94

Note: see more details in Supplementary Table S1.

The most frequently identified AE category for the three vaccines was the *behavioral and neurological AEs*, which includes 38 unique AEs (Table 3). Among them, 23 AEs were enriched in Pfizer vaccine, 22 were enriched in Janssen vaccine, and 6 were enriched in Moderna vaccine. Ageusia was enriched among all three vaccines. Migraine, parosmia, anosmia, panic attack, facial paralysis, seizure, and head discomfort were enriched in Janssen and Pfizer vaccines. Fatigue, impaired work ability, and chills were enriched in both Janssen and Moderna vaccines. Bell’s palsy was the only behavioral and neurological AE that was only enriched between Pfizer and Moderna vaccines. Headache, eye pain, unconsciousness, unresponsiveness to stimuli, and sleep disorder were only enriched in the Janssen vaccine. AEs that were only enriched in Pfizer vaccine included dysgeusia, anxiety, nervousness, chest discomfort, chest pain, oropharyngeal pain, axillary pain, hypoesthesia oral, and lymph node pain. Limb discomfort was enriched only in Moderna vaccine.

Fourteen enriched AEs were classified as *cardiovascular AEs*, of which 10 in Pfizer vaccine, 9 were enriched in Janssen vaccine, and 1 in Moderna vaccine. Other than atrial fibrillation, which was enriched in both Moderna and Pfizer vaccines, all other enriched cardiovascular AEs were associated with only the Pfizer and/or Janssen vaccines. Myocardial infarction, cerebrovascular accident, pulmonary embolism, thrombosis, and deep vein thrombosis were enriched in both Pfizer and Janssen vaccines. Palpitations had the highest case report count of all cardiovascular AEs and is only enriched in Pfizer vaccine. Other AEs that are only enriched in Pfizer vaccine include hypertension, heart rate irregular, and increased heart rate. Lastly, decreased heart rate, epistaxis, contusion, and pulmonary thrombosis were only enriched in Janssen (Figure 2).

*Female reproductive system AEs* were primarily enriched in both Janssen and Pfizer vaccines, including menstrual disorder, dysmenorrhea, heavy menstrual bleeding and irregular menstruation. Only vaginal hemorrhage was enriched exclusively for the Pfizer vaccine.

Nine adverse events were classified as *Local* (injection-site) *AEs*, of which all of them were enriched in the Moderna vaccine. These AEs included injection-site pruritus, injection-site erythema, injection-site edema, and injection site-warmth. Only one local AE (injection-site pain) was also enriched in the Pfizer vaccine.

Enriched *Immune system AEs* included myocarditis, pericarditis, and lymphadenopathy, which were all only enriched in the Pfizer vaccine. COVID-19 pneumonia was the only immune system AE that was enriched in both Janssen and Pfizer vaccines.

One enriched adverse event (death) was classified as a serious adverse event. Death was only enriched in the Janssen vaccine. There were 919 VAERS case reports of death for the Janssen vaccine as of 31 December 2021. This represents 1.44% out of 63,741 Janssen AE reports in VAERS, and only 0.0052% of the 17,640,334 total Janssen vaccination doses in the U.S. as of 31 December 2021 (https://covid.cdc.gov/covid-data-tracker/#vaccinations_vacc-total-admin-rate-total). The PRR value for death in the Janssen vaccine was 2.02, which means that the case report frequency of death in Janssen vaccine VAERS case reports was 2.02 times the case report frequency of death for all the other vaccines in VAERS. It should be noted that VAERS includes any adverse events that occur after vaccination, regardless of whether they were caused by vaccination. Thus, further investigation is needed to determine the cause of these reported deaths and identify any potential trends or patterns.

Other enriched AEs were classified as ear, eye, investigation result abnormal, homeostasis, injury, musculoskeletal or connective tissue, nervous system, respiratory system, skin, or urinary system adverse events. *Ear AEs* included deafness, ear discomfort, hypoacusis, and tinnitus. *Eye AEs* included vision blurred and vision field defect. The one *Investigation result abnormal AE* was oxygen saturation decreased. *Homeostasis AEs* included edema limbs, pharyngeal edema, tongue edema, thirst, and hypoxia. The one *Injury AE* was head injury. *Musculoskeletal or connective tissue AEs* included muscle spasm and muscle tightness. Paresthesia oral was classified as a *Nervous system AE*. *Respiratory system AEs* included acute respiratory failure, dyspnea exertional, sinus congestion, and respiratory tract congestion. *Skin AEs* included flushing, hot flushes, hyperhidrosis, and pruritic rash AEs. The one *Urinary system AE* was acute kidney injury.

### Myocarditis AE and the Effect of Sex, Age, and Dose Number

Among all three COVID-19 vaccines, myocarditis was associated with 2,295 cases as of 31 December 2022. Specifically, the Janssen vaccine had 73 cases of myocarditis (0.11% case report frequency) (Table 4). The Pfizer vaccine had 1,471 (0.46%), while the Moderna vaccine had 751 cases (0.23%). Of the three vaccines, the Pfizer vaccine had the highest case report frequency of myocarditis, and it was the only vaccine for which myocarditis was significantly enriched according to our statistical criteria. However, the low frequency of myocarditis among all case reports for the Pfizer vaccine indicates that it is a rare AE: out of 1,000 Pfizer vaccine-related AE case reports to VAERS, there were fewer than 5 cases reporting myocarditis.

**TABLE 4:** Case report counts, frequencies, and PRRs of 3 representative AEs for the Janssen, Moderna, and Pfizer vaccines. Bolded values represent statistically significant AEs for the corresponding vaccine.

	Janssen Vaccine (cases, %, PRR)	Pfizer Vaccine (cases, %, PRR)	Moderna Vaccine (cases, %, PRR)
Total	63,801	322,281	329,457
GBS	233 (0.37%, 1.29)	340 (0.11%, 0.31)	243 (0.07%, 0.21)
Myocarditis	73 (0.11%, 0.62)	**1,471 (0.46%, 4.34)**	751 (0.23%, 1.35)
Thrombosis	**992 (1.56%, 6.84)**	**1,708 (0.53%, 2.45)**	1,196 (0.36%, 1.38)

The bold values are the statistically significant case report frequency and PRR.

**FIGURE 3:**
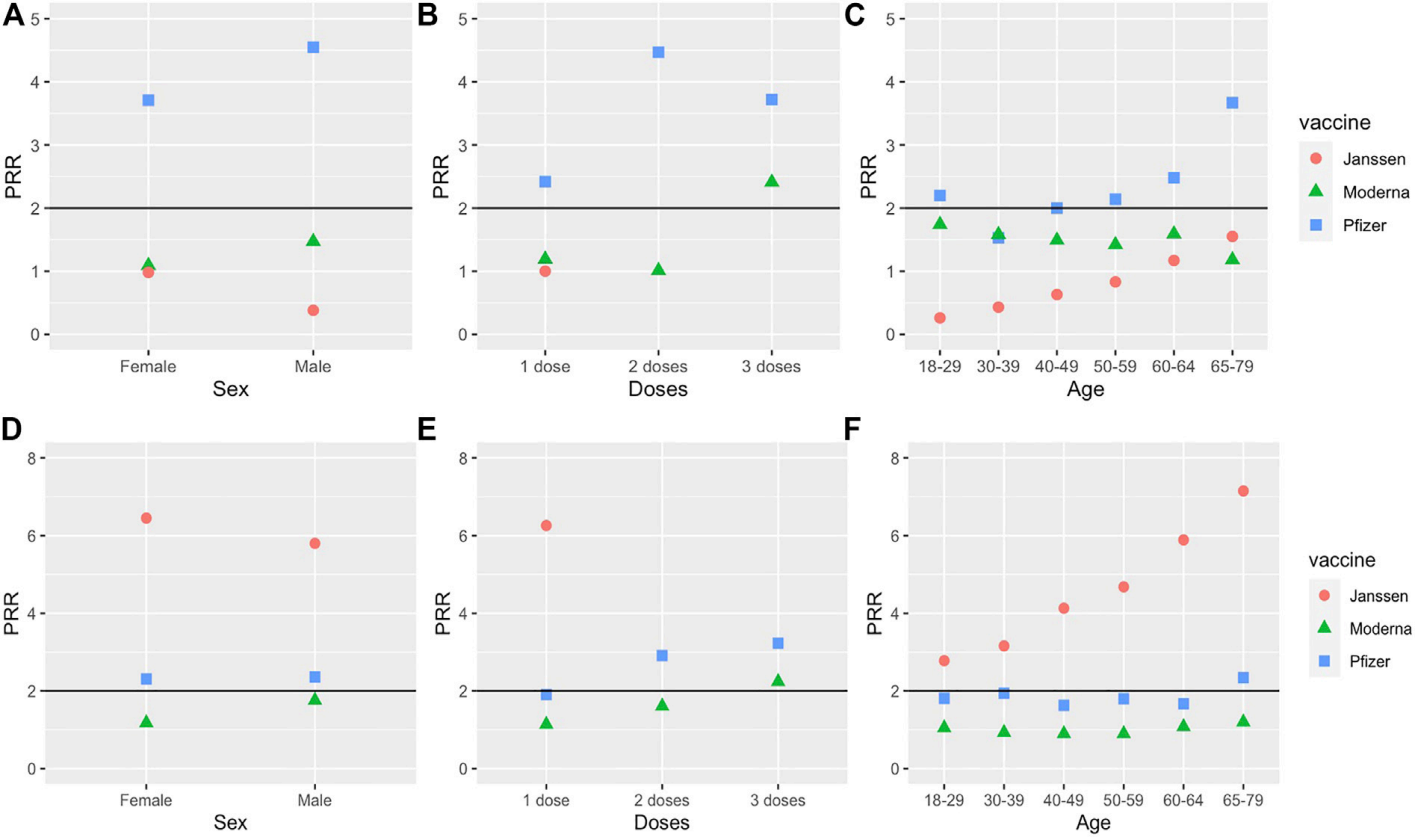
PRRs of myocarditis and thrombosis under 3 conditions (sex, dose number, and age). **(A–C)** are for myocarditis, and **(D–F)** are for thrombosis. The effects of sex, dose, and age are studied. Note that we only used Dose 1 data for the Janssen vaccine.

Statistical results for each specific sex, age group, and dose number are found in Supplementary File S3. For both males and females, the PRR for myocarditis in the Pfizer vaccine was significant, but not for the Moderna or Janssen vaccines (Figure 3A). Myocarditis after Pfizer vaccination also had significant Chi-square statistics for both sexes (Supplementary File S3). However, the case report frequency of myocarditis in Pfizer was significant for males but not females. Thus, myocarditis was significantly enriched in the Pfizer vaccine for males but not females. For both the Moderna and Janssen vaccines, myocarditis did not meet the significance criteria for either males or females.

The results of different doses of the three vaccines were compared as well. Myocarditis had significant PRR values for all three doses of Pfizer and dose 3 of Moderna, and these doses also met the significance criteria for Chi-square statistic and case report frequency (Figure 3B). Of the three Pfizer doses, Dose 2 of the Pfizer vaccine had the highest PRR value (4.47). Doses 1 and 2 of the Moderna vaccine and Dose 1 of the Janssen vaccine failed to meet at least one of the three significance criteria.

The results for different age groups were also compared. Myocarditis met the significance criteria for age groups 18–29 and 40–49 receiving the Pfizer vaccine. While the PRR for myocarditis did meet the significance criteria for age groups 50–59, 60–64, and 65–79 receiving the Pfizer vaccine, the case report frequency of myocarditis AE for these groups was less than 0.2% (Figure 3C). Myocarditis was not significant for any age group receiving the Moderna or Janssen vaccines.

### GBS AE and the Effect of Sex, Age, and Dose Number

Although GBS has received much attention in the news, it was not significantly enriched in any of the three vaccines. As of 31 December 2021, there were 233 (0.37% case report frequency), 340 (0.11%), and 243 (0.07%) GBS case reports associated with Janssen, Pfizer, and Moderna vaccines, respectively (Table 4). The case report frequency of the Janssen vaccine-associated GBS cases was 3.46 times over that of Pfizer vaccine and 4.95 times that of Moderna vaccine (Table 4). However, the PRR values of the three COVID-19 vaccines (Janssen: 1.29; Pfizer: 0.312; Moderna: 0.212) were all less than 2, and were not significant based on our filtering criteria.

The case report frequency of GBS was significant (>0.2%) for all sexes, Dose 1, and all age groups except for age group 18–29 for the Janssen vaccine (Supplementary File S3). However, no sexes, doses, or age groups for the Moderna or Pfizer vaccines had a significant case report frequency for GBS. The Chi-square statistics for GBS were significant (>4) for both males and females for the Pfizer and Moderna vaccines, but were not significant for either sex for the Janssen vaccine. The Chi-square statistics for GBS were also significant for all age groups for the Pfizer and Moderna vaccines and for the age groups 18–29, 50–59, and 60–64 for the Janssen vaccine. Dose 1 for all three vaccines, Dose 2 for the Pfizer vaccine, and Dose 3 for the Pfizer and Moderna vaccines had significant Chi-square statistics. The PRR values of GBS for all sexes, doses, and age groups for all 3 vaccines failed to meet the significance criteria of >2, except for Dose 2 for the Moderna vaccine. Overall, GBS failed to meet one or more of the significance criteria for the Janssen, Pfizer, and Moderna vaccines for all sexes, doses, and age groups.

### Thrombosis AE and the Effect of Sex, Age, and Number of Doses

As shown in Table 4, there were 992 (1.56% case report frequency), 1,708 (0.53%), and 1,196 (0.36%) thrombosis cases associated with Janssen, Pfizer, and Moderna vaccines as of the end of 2021. Thrombosis met all three significance criteria for the Janssen and Pfizer vaccines, but not Moderna. The Janssen vaccine-associated thrombosis AE had a PRR value of 6.84, indicating that thrombosis was reported more than 6 times as frequently for the Janssen vaccine compared to all the other vaccines in VAERS. Meanwhile, the thrombosis AE associated with the Pfizer and Moderna vaccines had a PRR value of 2.45 and 1.38 (Table 4), respectively, suggesting that these two vaccines were less likely associated with the thrombosis AE.

Thrombosis had significant PRR values for both females and males receiving the Pfizer and Janssen vaccines (Figure 3D). For the Janssen vaccine, the PRR for females was slightly greater compared to males. The PRR values for males and females receiving the Janssen vaccine were both greater than the PRR values for males and females receiving the Pfizer vaccine. Thrombosis also had significant case report frequencies and Chi-square statistics for both males and females receiving the Pfizer or Janssen vaccines. However, thrombosis failed to meet at least 1 of the significance criteria for both males and females receiving the Moderna vaccine.

Thrombosis was significant for Dose 1 of the Janssen vaccine, Dose 2 of the Pfizer vaccine, and Dose 3 of the Pfizer and Moderna vaccines (Supplementary File S3). Notably, the thrombosis PRR for Dose 1 of the Janssen vaccine (6.26) was considerably higher than the PRRs for other doses for any COVID-19 vaccine. Dose 1 of the Pfizer vaccine met the significance criteria for case report frequency and Chi-square statistic, but its PRR of 1.91 was slightly below the significance cutoff of 2.

Thrombosis was significant for all six age groups of the Janssen vaccine, as well as the 65–79 age group for the Pfizer vaccine. However, thrombosis was not significant for any age group of the Moderna vaccine. Interestingly, the PRR values for thrombosis in the Janssen vaccine appear to increase with age (Figure 3F).

### OAE Ontology Updates and Modeling to Support COVID-19 Vaccine AE Research

As a community-based ontology in the field of adverse events, the Ontology of Adverse Events (OAE) standardizes the definition and classification of various adverse events (He et al., 2014). The thrombosis with thrombocytopenia syndrome (TTS) (Wise et al., 2007) is a new adverse event recently proposed during the COVID-19 pandemic. This term is not present nor defined in the MedDRA AE coding system. As the OAE developers by ourselves, we have updated OAE by providing a standardized definition of the term ‘thrombosis with thrombocytopenia syndrome AE’ in the OAE. Our OAE definition on this TTS term is based on the term standardization by the Brighton Collaboration (Brighton Collaboration, 2021).

**FIGURE 4:**
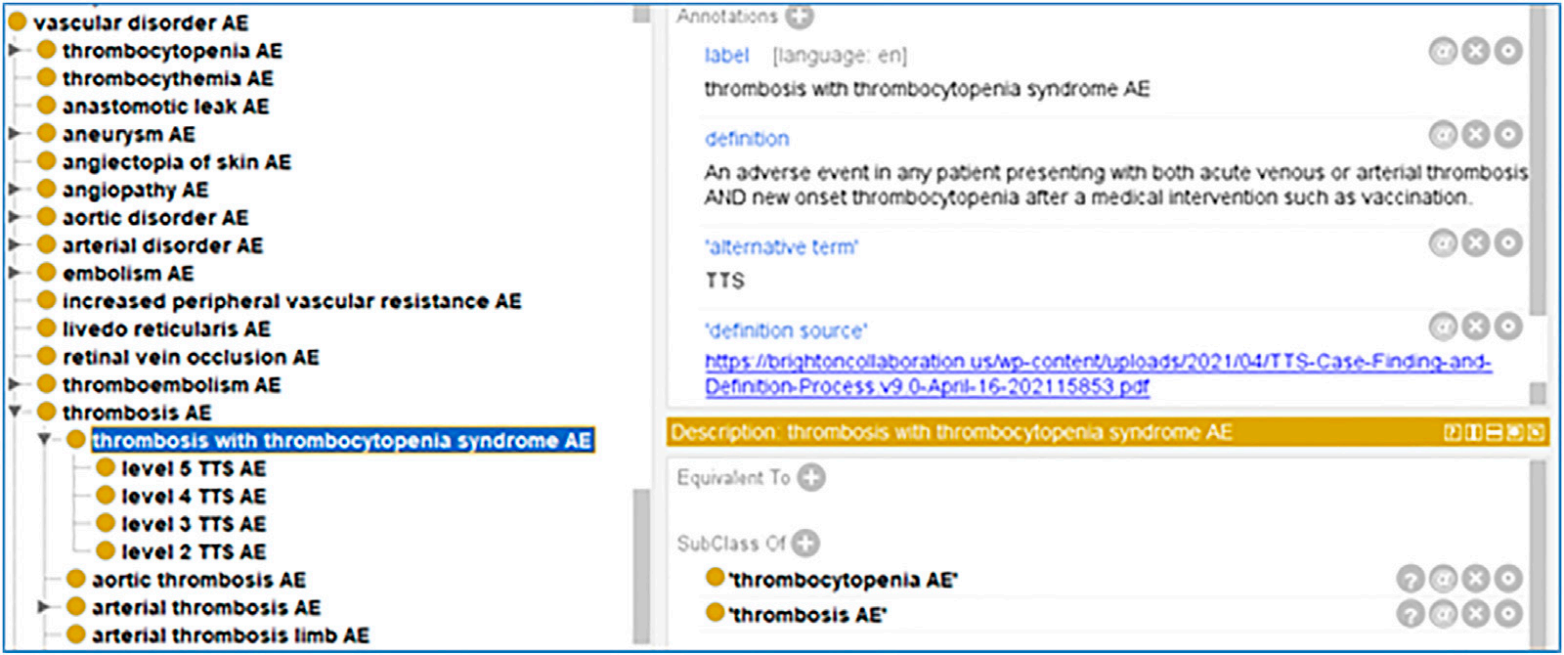
OAE definition of thrombosis with thrombocytopenia syndrome (TTS) AE and its five level subtypes based on Brighton Collaboration case definition (Chen and Black, 2021).

Based on the Brighton Collaboration definition, we also defined the term in the OAE. Given that OAE focuses on adverse events, we named our term ‘thrombosis with thrombocytopenia syndrome AE’ and defined it as: “An adverse event in a patient presenting with both acute venous or arterial thrombosis and new onset thrombocytopenia after a medical intervention such as vaccination” (Figure 4).

It is noted that the presence of both thrombosis and thrombocytopenia does not mean TTS per se because they might have been assessed separately. To make a TTS case established, both thrombosis and thrombocytopenia should exist together. Since an exposure to heparin might induce thrombocytopenia (Greinacher et al., 2021), any recent known exposure to heparin should be excluded in the conclusion of TTS adverse event following vaccination.

Furthermore, based on the Brighton Collaboration’s recommendation, we have classified five levels of TTS AE (Chen and Black, 2021), and include these five levels of TTS AE to OAE (Figure 4). These five levels are briefly classified as follows (Chen and Black, 2021):

- Level 1 TTS AE: definite case of TTS.
- Level 2 TTS AE: probable case of TTS.
- Level 3 TTS AE: possible case TTS.
- Level 4 TTS AE: reported case as TTS but having insufficient evidence.
- Level 5 TTS AE: most likely not a case of TTS

The above OAE-represented AE terms can be used for COVID-19 VAE or AE representation. To further represent the semantic relations between the AEs and specific COVID-19 vaccines, we used the Coronavirus Infectious Disease Ontology (CIDO) (He et al., 2020; Huffman et al., 2021; Liu et al., 2021) as the platform. As a biomedical ontology in the domain of coronavirus diseases, the CIDO has also imported the terms of COVID-19 vaccine-related terms from the Vaccine Ontology (VO) (Ozgur et al., 2011; Lin and He, 2012). As an example of how an ontological relation can be used to associate a vaccine with an adverse event, we linked the Janssen vaccine and the TTS AE using the relation ‘*vaccine susceptible for AE*’, we were able to link a COVID-19 vaccine and its associated AE such as:

“Janssen vaccine”: “vaccine susceptible for AE” some “TTS AE.”

Note that not all the statistically identified AEs found in this study were included in the CIDO representation. Only clinical or experimentally verified AEs are included. Ontology is a foundation of AI. Through an ontological representation such as CIDO’s, adverse event data can be understood and interpreted by various AI programs, supporting advanced AI reasoning and studies.

## Discussion

VAERS has been widely used for COVID-19 vaccine AE studies, and similar results have been reported (Chen et al., 2021; Singh et al., 2022). For example, Singh’s study found that the most common AE for the three vaccines is headache and most of the AEs are mild (Singh et al., 2022). Compared to other VAERS-based studies, our work is unique in at least three aspects. First, we have applied a standardized statistical methodology incorporating Pearson’s Chi-square statistic, PRR, and minimal case report number to identify enriched AEs. Second, we applied our Ontology of Adverse Events (OAE) classification methods to classify statistically enriched AEs into various categories, including behavioral and neurological, cardiovascular, renal, pulmonary, reproductive, and skin AEs. Third, we have also generated a publicly available database of COVID-19 vaccine VAERS case reports specifically for COVID-19 vaccines using the COVID-19 Vaccine Knowledge Base web platform (Cov19VaxKB) (Huang et al., 2021), and these case reports can be queried and further analyzed using our web interface.

Our study found that the number of enriched AEs for the three vaccines has shifted considerably over time (Supplementary Figure S1). From April 2021 to December 2021, the number of statistically significant AEs shared across all three COVID-19 vaccines decreased from 44 to 3 while the number of enriched AEs shared between the Pfizer and Janssen vaccines increased from 10 to 21. Notably, the number of significantly enriched AEs associated with the Moderna vaccine sharply decreased from 94 in April 2021 to 33 in December 2021. These changes could be attributed to the influx of new case reports over time that provided stronger statistical power to our VAERS analysis. Meanwhile, other factors such as number of doses and possible earlier infection might have also affected the results.

Vaccinated females appeared to have higher case report frequencies than vaccinated males for the top 10 reported COVID-19 vaccine AEs (Table 2). Our finding is largely aligned with a recent report that women have a wider range of AEs to experience than men after COVID-19 vaccination (Venkatakrishnan et al., 2021). According to that report, the women were more susceptible to AEs such as nausea, vomiting, erythema, local pain and swelling, headache, diarrhea, and anaphylaxis, and men were more susceptible to AEs including fever, facial paralysis, and myalgia after COVID-19 vaccine administration. However, our study showed that the females were more susceptible to all top 10 VAEs, including fever (i.e., pyrexia) and myalgia AEs, than the males based on the VAERS data analysis. Females typically develop higher antibody responses than males, rendering not only more resistance of females to infectious diseases but also higher incidence to autoimmunity and adverse events among women (Fischinger et al., 2019). Others possible factors that could explain higher AE occurrences in females include sex differences in hormones, genetics, microbiome profiles, and sensitivity to pain (Venkatakrishnan et al., 2021). Confounding factors such as prior history of COVID and willingness to reporting to VAERS might also have effects on the sex differences observed in our study.

Among enriched behavioral and neurological AEs in at least one of the three COVID-19 vaccines, Bell’s palsy (i.e., unexplained facial muscle weakness or paralysis), ageusia (i.e., the loss of taste), dysgeusia (i.e., taste distortion), and anosmia (i.e., loss of smell) are worth discussion. Bell’s palsy was enriched in both Moderna and Pfizer vaccines. Prior population-based studies suggest that the risk of Bell’s palsy following Pfizer vaccination is either negligible or slightly increased (Shibli et al., 2021; Wan et al., 2022). A population-based case-control study of COVID-19 vaccine recipients in Hong Kong found no significantly increased risk of Bell’s palsy in Pfizer vaccine recipients (Shibli et al., 2021). However, a retrospective cohort study based on healthcare data from Israel concluded that the Pfizer vaccine may be associated with an increased risk of Bell’s palsy (Wan et al., 2022).

Notably, taste disorder and ageusia were the only AEs that were significantly enriched among all three vaccines. Case studies have been reported for loss of taste following COVID-19 vaccination, but further investigation is needed to determine any large-scale patterns or a causal relationship between COVID-19 vaccination and loss of taste (Lechien et al., 2021).

Cardiovascular AEs have been frequently associated with COVID-19 vaccines. Most of the enriched cardiovascular-related AEs were only enriched in the Pfizer and/or Janssen vaccines, including myocarditis and thrombosis (discussed below). However, atrial fibrillation was enriched in the Pfizer and Moderna vaccines. Although there have not been any studies that have further investigated any relationship between atrial fibrillation and SARS-CoV-2 vaccination, there have been studies that have described occurrences of arrhythmia following administration of the Pfizer COVID-19 vaccine. For example, there have been a few cases of paroxysmal ventricular arrhythmia in the BNT162b2 (Pfizer) vaccine clinical trial; however, no causality was able to be established (Polack et al., 2020). There have also been studies reporting multiple cases of tachycardia post vaccination (Marco Garcia et al., 2021). Other studies have also observed occurrences of myocardial infarction after COVID-19 vaccination, with a higher rate of myocardial infarction associated with older people (Li X. et al., 2021). Although isolated case reports have been identified for the aforementioned AEs, further population-based, longitudinal studies are needed to establish a causal relationship between cardiovascular AEs and COVID-19 vaccination.

The increasing number of *de novo* or reactivation of acute kidney injury (AKI) has been reported after COVID-19 vaccination. The occurrence of AKI after immunization against influenza, pneumococcus, and hepatitis B has been accounted for previously (Hou et al., 2021). A review of AKI-related case reports after COVID-19 vaccination found that the most frequent pathology observed in patients who developed acute onset nephrotic syndrome was minimal change disease (MCD, 19 cases), followed by IgA nephropathy (14 cases) and vasculitis (10 cases). In our study, we found AKI was significantly enriched in Janssen and Pfizer. Given that AKI is known to occur as a side effect for various other vaccines, it is therefore no surprise that it is enriched as an adverse event in the Janssen and Pfizer vaccines.

A few enriched AEs were related to the female reproductive system, including irregular menstruation, heavy menstrual bleeding, and intermenstrual bleeding. A January 2022 retrospective cohort study tracking menstrual cycle data from nearly 4,000 vaccinated and unvaccinated individuals found that COVID-19 vaccination was associated with a change of less than 1 day in menstrual cycle length (Edelman et al., 2022). This change is considered clinically insignificant since it falls within the normal variation for cycle length (less than 8 days) according to the International Federation of Gynecology and Obstetrics. This study also found no association between vaccination and change in menses length. As for heavy menstrual bleeding or intermenstrual bleeding, no studies discussing the relation of these adverse events to COVID-19 vaccination were found. Shimabukuro et al. found that the overall reactogenicity profile was similar between pregnant and non-pregnant women (Hause et al., 2021).

As suggested by the CDC, myocarditis, GBS, and thrombosis are three medical conditions that might be associated with Pfizer, Moderna, and/or Janssen COVID-19 vaccines (https://www.cdc.gov/mmwr/volumes/70/wr/mm7032e4.htm). Our VAERS case report analysis did not find GBS as a significantly enriched adverse event in any of the three vaccines but did find that myocarditis was enriched in the Pfizer vaccine, while thrombosis was enriched in both the Pfizer and Janssen vaccines. In light of these results, we performed a more thorough statistical analysis of GBS, thrombosis, and myocarditis in VAERS case reports related to these three vaccines based on sex, age, and number of doses.

Our findings regarding myocarditis are similar to some prior studies. Li et al. conclude that the rate of myocarditis/pericarditis is low. Among all vaccines in their study, the rate is the highest in BNT162b2, particularly in the second dose (Li M. et al., 2021). According to Aye et al., many of those who developed myocarditis had symptoms after the second dose (Aye et al., 2021). We also found that the results of the Pfizer vaccine (BNT162b2) were significant after two doses. Furthermore, Dionne et al. conducted case studies and stated that myocarditis occurred more frequently in male after the second dose of BNT162b2 (Dionne et al., 2021).

The PRR of Pfizer for myocarditis was the highest among all three vaccines in our study, suggesting that the second dose after the vaccination might be riskier, especially for the Pfizer vaccine, compared to other doses and vaccines. Our findings regarding myocarditis are similar to some prior studies. An analysis of VAERS data from 11 December 2020, to 13 August 2021, concluded that while the rate of myocarditis and pericarditis after COVID-19 vaccination is low, these adverse events occur most frequently in the Pfizer COVID-19 vaccine, particularly after the second dose (Li M. et al., 2021). Other studies have also suggested that post-vaccination incidents of myocarditis occurred more frequently after the second dose of the Pfizer vaccine compared to the first dose (Aye et al., 2021; Dionne et al., 2021). Our results also indicated that myocarditis was significantly enriched in the Pfizer vaccine for males and adults aged 18–29. A study analyzing safety surveillance data from Vaccine Safety Datalink as of 26 June 2021, suggested that the association of myocarditis with the Pfizer vaccine was elevated, though not significant, in people of ages 12 to 39 (Klein et al., 2021).

We did not find a significant statistical association between GBS and any of the three COVID-19 vaccines. Many case reports of GBS after COVID-19 vaccination have been published (Supplementary File S1). For example, Maramattom et al. observed seven cases of GBS that occurred within 2 weeks of the first dose of ChAdOx1-S vaccination (Maramattom et al., 2021). Caress et al. reviewed 37 published cases of GBS associated with COVID-19 during the early pandemic and found a potentially small but statistically significant safety issue with GBS following Ad26.COV2.S vaccine (Caress et al., 2020). Nagalli and Kikkeri reported the first known case of subacute GBS onset following mRNA-1273 vaccination, in which a middle-aged woman gradually developed lower extremity weakness, symptoms peaked at 10 and 12 weeks after onset (Nagalli and Shankar Kikkeri, 2022). Roberto et al. reported a case of GBS after receiving the second dose of Pfizer vaccine (Scendoni et al., 2021). GBS has also previously been observed as an adverse event of several vaccines that incorporate viral genetic material (Leung, 2021; Oliver et al., 2022). However, no significant statistical association between GBS and COVID-19 vaccines was observed in our study. Two possible reasons might explain this finding. First, additional data uploaded to VAERS since the publication of these earlier studies may have influenced the results of our GBS case report analysis and contributed to greater statistical power for our analysis, since we analyzed a much higher sample size of case reports (816 vs. 37 or fewer). Second, the statistical methods used are different. Caress et al. analyzed the observed ratios and considered an increase in the percentages to be statistically significant (Caress et al., 2020). However, our study utilizes PRR, Chi-square, and case report frequencies to determine significantly enriched adverse events.

Thrombosis was found significant for the Janssen and Pfizer vaccines. Our finding that the Janssen vaccine was significant for thrombosis is not surprising since the Janssen vaccine is an adenoviral vector vaccine, which is a type of vaccine known to be correlated with thrombosis with thrombocytopenia syndrome (TTS) (Ashorobi et al., 2022). However, it is notable that the Pfizer vaccine was also significant for thrombosis, since prior studies have suggested that there is no association between thrombosis or TTS with mRNA vaccines such as the Pfizer and Moderna vaccines (Klein et al., 2021). Further studies are necessary to investigate the potential link between thrombosis and the Pfizer COVID-19 vaccine.

The molecular mechanisms underlying these enriched AEs merit further investigation. Recent research has provided evidence of the antibody-mediated response to molecular complexes between platelet factor 4 (PF4) and the adenoviral vectors used in COVID-19 vaccines, which appears to be the likely mechanism of the unusual blood clot occurrence associated with adenoviral vector-based COVID-19 vaccines (Baker et al., 2021). The binding of the antibodies to the complex was able to induce platelet activation. Several studies have observed associations between vaccinee comorbidities and VAE outcomes. For example, Chen et al. found that the history of allergy was more frequent in people with anaphylactic reactions as AEs, and the history of anxiety or depression was more common in people with severe neurological VAEs after vaccination (Chen et al., 2021). Those who died were likely to be elderly and were more prone to experience hypertension and neurological disorders (Chen et al., 2021). Xiong’s study found that the adverse events for older adults are more serious than younger adults according to the results of logistic regression models (Xiong et al., 2021). Further studies are necessary to determine whether these and other comorbidities may be causally linked to specific adverse events in COVID-19 vaccines.

We also reviewed studies analyzing COVID-19 vaccine AE case reports from other databases including the EudraVigilance Database and VigiBase (Supplementary File S1). A study analyzing data from the EudraVigilance Database found that the frequencies of thrombocytopenia and severe AEs in young adult recipients were lower in Janssen compared to the AstraZeneca COVID-19 vaccine; however, the frequency of severe AEs in older recipients was higher in Janssen compared to AstraZeneca (Cari et al., 2021). As part of the European Monetary System (EMA), the Pharmacovigilance Risk Assessment Committee stated that unusual thrombosis with low blood platelets is a rare adverse event of Janssen (Douxfils et al., 2021), which agrees with our results. EMA also reported a serious and rare adverse effect of thrombosis with thrombocytopenia syndrome (TTS) after the vaccination of ChAdOx1 nCoV-19 in March 2021 (See et al., 2021). An analysis of VigiBase adverse event case reports for the Pfizer, AstraZeneca, and Moderna COVID-19 vaccines found that acute myocardial infarction, cardiac arrest, and circulatory collapse were associated with these vaccines in people over 75 years old (Jeet Kaur et al., 2021).

Our statistical analysis has identified a number of AEs that are significantly enriched in COVID-19 vaccines compared to all vaccines in the VAERS database. However, VAERS data still presents limitations due to the passive reporting nature of this database (Shimabukuro et al., 2015). The VAERS raw data submitted by the original submitters is processed through a standardization step using the MedDRA coding system (Shimabukuro et al., 2015). After this initial processing, VAERS case reports are made available for public viewing via CDC Wonder (Rodriguez-Nava et al., 2020) or direct download. These case reports are constantly monitored for duplicates or false information and are removed when such information is detected (https://www.cdc.gov/vaccinesafety/ensuringsafety/monitoring/vaers/index.html). However, it is possible that case reports may contain “incomplete, inaccurate, coincidental and unverified information” (https://vaers.hhs.gov/data.html). Underreporting of adverse event incidents has also been cited as a potential pitfall of the VAERS system (Shimabukuro et al., 2015). This suggests that the true number of AE cases for COVID-19 vaccines may be greater than the crude AE reporting rates that were calculated in this study.

As stated in the VAERS disclaimer, vaccination may not necessarily be the cause of AEs reported in the system (https://vaers.hhs.gov/data.html). With respect to COVID-19 vaccines in particular, comparison with VAERS case reports for other vaccines has limitations since COVID-19 vaccine data as of 31 December 2021, was primarily derived from adults, whereas other vaccines may be mainly administered to children. Factors that could affect the incidence of adverse events and increase variability in case reports include prior COVID-19 infection and preexisting comorbidities. Therefore, we cannot identify a causal relationship between COVID-19 vaccination and a specific adverse event based on VAERS data even if a statistically significant association is observed. Our findings require further investigation to determine if a causal relationship between COVID-19 vaccination and these enriched adverse events exists.

Overall, most COVID-19 vaccine-associated AEs occur very rarely, even those that were significantly enriched in specific COVID-19 vaccines. As of 31 December 2021, a total of 509,307,789 COVID-19 vaccines were administered in the United States, while 717,577 adverse event case reports were collected in VAERS (https://covid.cdc.gov/covid-data-tracker/#vaccinations_vacc-total-admin-rate-total). These values equate to a crude AE reporting rate of 0.14% for all COVID-19 vaccines. The most frequent AEs reported among the three COVID-19 vaccines were relatively mild effects such as headache (17.5% of all reports), pyrexia (14.8%), fatigue (14.6%), and chills (12.7%). More severe AEs that were significantly enriched in at least one COVID-19 vaccine occurred at relatively low frequencies. For instance, myocarditis, which was enriched in the Pfizer vaccine, had 1,542 VAERS case reports for the Pfizer vaccine. Similarly, thrombosis, which was enriched in the Pfizer and Janssen vaccines, had a case report frequency of 1,737 for Pfizer and 1,004 for Janssen. It is worthy to note that myocarditis and thrombosis have also been associated with SARS-CoV-2 infection. Studies have found that the risk of myocarditis, pericarditis, cardiac arrhythmias, thrombocytopenia, and thromboembolism is much greater after SARS-CoV-2 infection compared to after COVID-19 vaccination (Hippisley-Cox et al., 2021; Patone et al., 2021). While our VAERS case report analysis has identified signals and alarms for possible adverse events associated with COVID-19 vaccines, the existing COVID-19 vaccines are generally very safe and the benefits of vaccination outweigh the risks.

## Data Availability

The Ontology of Adverse Events (OAE) source code is available at the OAE GitHub website: https://github.com/OAE-ontology/OAE/.

## Supplementary Material

Supplementary Figure S1 Results of statistically enriched AEs obtained at different time points from VAERS for the Janssen, Moderna, and Pfizer vaccines. **(A)** Data as of April 9, 2021, **(B)** as of May 26, 2021, **(C)** as of October 29, 2021, and **(D)** as of Dec 31, 2021. The number of shared AEs appeared to decrease over time with increasing sample sizes.

Supplementary File S1 All literature data of human adverse events (AEs) associated with myocarditis, GBS, thrombosis, and other AEs. This Excel file includes four spreadsheets that include the literature data associated with general enriched AEs, myocarditis, GBS, and thrombosis, respectively.

Supplementary File S2 List of significant adverse events for the Pfizer, Moderna, and Janssen vaccines before and after trimming for medically relevant AEs. Text output for the Venn Diagrams of enriched AEs for the Janssen, Moderna, and Pfizer vaccines is also included. Case report frequencies, PRR, and Chi-square statistics are included for non-trimmed enriched AEs. Note that different spreadsheets are included in this Excel file.

Supplementary File S3 Case report frequencies, PRR, and Chi-square statistics for the myocarditis, GBS, and thrombosis adverse events in the Janssen, Moderna, and Pfizer vaccines for specified age, sex, and dosage groups.

Supplementary Table S1 Case report counts and frequencies, crude reporting rates (per 1 million doses), PRR, and Chi-square statistics for AEs that are significant for at least one COVID-19 vaccine.
