# Supplementary material for: Profiling COVID-19 Vaccine Adverse Events by Statistical and Ontological Analysis of VAERS Case Reports
Source: Front Pharmacol. 2022 Jun 24;13:870599. doi: 10.3389/fphar.2022.870599 (PMC9263450; doi:10.3389/fphar.2022.870599)
Supplement: Supplementary file 1 [file DataSheet1.zip › Supplemental Figure S1.DOCX]

**
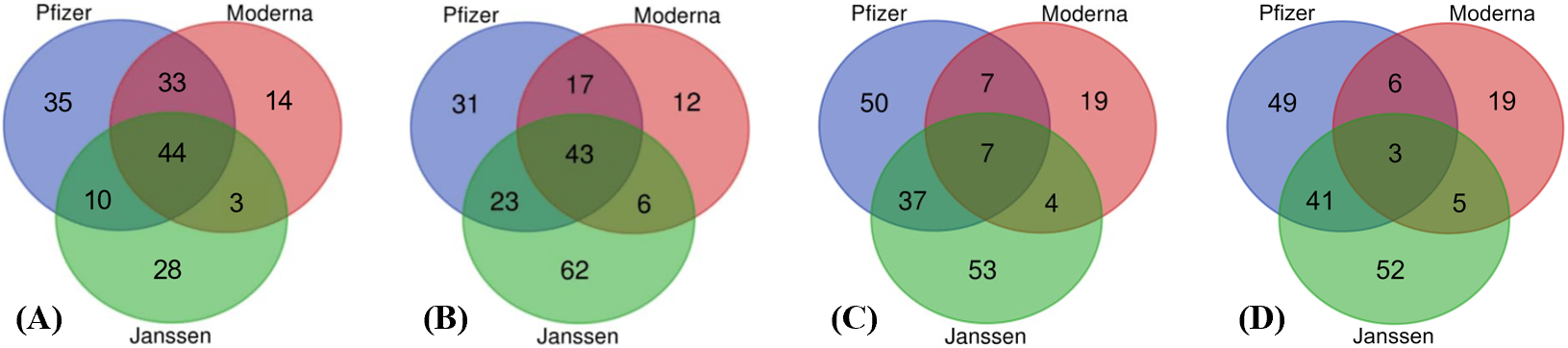
**

**Supplemental Figure 1. Results of statistically enriched AEs obtained at different time points from VAERS for the Pfizer, Moderna, and Janssen vaccines. (A)** Data as of April 9, 2021. **(B)** Data as of May 26, 2021. **(C)** Data as of October 29, 2021. and **(D)** Dec 31, 2021. The number of shared AEs appeared to decrease over time with increasing sample sizes.
