# Supplementary material for: Profiling COVID-19 Vaccine Adverse Events by Statistical and Ontological Analysis of VAERS Case Reports
Source: Front Pharmacol. 2022 Jun 24;13:870599. doi: 10.3389/fphar.2022.870599 (PMC9263450; doi:10.3389/fphar.2022.870599)
Supplement: Supplementary file 1 [file DataSheet1.zip › Supplemental Table 1.DOCX]

**Supplemental Table 1.** Case report counts and frequencies, crude reporting rates (per 1 million doses), PRR, and Chi-squared values for AEs that are significant for at least one COVID-19 vaccine (significant vaccines for each AE are **bolded**).

| **Adverse Event** | **Vaccine** | **Counts** | **Frequency (%)** | **Reporting Rate (per 1 million doses)** | ***PRR*** | 𝑥^2^ |
| --- | --- | --- | --- | --- | --- | --- |
| ***Behavioral and neurological AE*** (38) | | | | | | |
| ageusia | **Janssen** | 439 | 0.69 | 24.89 | 2.13 | 241.48 |
|  | **Moderna** | 1866 | 0.57 | 9.61 | 2.08 | 654.59 |
|  | **Pfizer** | 2381 | 0.74 | 8.02 | 3.26 | 1944.30 |
| anosmia | **Janssen** | 344 | 0.54 | 19.50 | 2.18 | 199.33 |
|  | Moderna | 1357 | 0.41 | 6.99 | 1.90 | 375.30 |
|  | **Pfizer** | 2007 | 0.62 | 6.76 | 3.92 | 2070.10 |
| anxiety | Janssen | 833 | 1.31 | 47.22 | 1.78 | 266.38 |
|  | Moderna | 2918 | 0.89 | 15.02 | 1.23 | 93.01 |
|  | **Pfizer** | 4279 | 1.32 | 14.41 | 2.21 | 1763.70 |
| cold sweat | **Janssen** | 659 | 1.03 | 37.36 | 2.50 | 535.31 |
|  | Moderna | 1610 | 0.49 | 8.29 | 1.15 | 23.12 |
|  | Pfizer | 1996 | 0.62 | 6.72 | 1.58 | 297.07 |
| dysgeusia | Janssen | 566 | 0.89 | 32.09 | 1.69 | 148.58 |
|  | Moderna | 2667 | 0.81 | 13.73 | 1.75 | 571.01 |
|  | **Pfizer** | 3391 | 1.05 | 11.42 | 2.64 | 1985.12 |
| fatigue | **Janssen** | 10913 | 17.12 | 618.64 | 2.01 | 5538.40 |
|  | **Moderna** | 47860 | 14.54 | 246.37 | 2.00 | 16620.00 |
|  | Pfizer | 45517 | 14.08 | 153.32 | 1.89 | 13693.00 |
| impaired work ability | **Janssen** | 656 | 1.03 | 37.19 | 2.20 | 393.88 |
|  | **Moderna** | 2714 | 0.82 | 13.97 | 2.09 | 964.26 |
|  | Pfizer | 2572 | 0.80 | 8.66 | 1.96 | 785.57 |
| nervousness | Janssen | 211 | 0.33 | 11.96 | 1.50 | 32.90 |
|  | Moderna | 829 | 0.25 | 4.27 | 1.16 | 13.07 |
|  | **Pfizer** | 1212 | 0.38 | 4.08 | 2.05 | 411.43 |
| night sweats | **Janssen** | 314 | 0.49 | 17.80 | 2.04 | 151.81 |
|  | Moderna | 1260 | 0.38 | 6.49 | 1.78 | 284.49 |
|  | Pfizer | 1155 | 0.36 | 3.89 | 1.60 | 179.51 |
| panic attack | **Janssen** | 139 | 0.22 | 7.88 | 2.13 | 76.22 |
|  | Moderna | 462 | 0.14 | 2.38 | 1.44 | 43.31 |
|  | **Pfizer** | 672 | 0.21 | 2.26 | 2.64 | 391.61 |
| Bell's palsy | Janssen | 275 | 0.43 | 15.59 | 1.85 | 98.62 |
|  | **Moderna** | 1384 | 0.42 | 7.12 | 2.21 | 558.43 |
|  | **Pfizer** | 1728 | 0.53 | 5.82 | 3.36 | 1467.30 |
| facial paralysis | **Janssen** | 274 | 0.43 | 15.53 | 2.10 | 144.77 |
|  | Moderna | 962 | 0.29 | 4.95 | 1.53 | 120.88 |
|  | **Pfizer** | 1333 | 0.41 | 4.49 | 2.61 | 761.59 |
| seizure | **Janssen** | 650 | 1.02 | 36.85 | 2.50 | 527.36 |
|  | Moderna | 1603 | 0.49 | 8.25 | 1.16 | 27.10 |
|  | **Pfizer** | 2373 | 0.73 | 7.99 | 2.10 | 860.96 |
| chills | **Janssen** | 11387 | 17.86 | 645.51 | 2.31 | 8299.80 |
|  | **Moderna** | 12512 | 3.80 | 64.41 | 19.19 | 15573.00 |
|  | Pfizer | 35254 | 10.91 | 118.75 | 1.47 | 4082.4 |
| chest discomfort | Janssen | 1292 | 2.03 | 73.24 | 1.57 | 255.82 |
|  | Moderna | 5653 | 1.72 | 29.10 | 1.43 | 516.58 |
|  | **Pfizer** | 8180 | 2.53 | 27.55 | 2.59 | 4673.10 |
| head discomfort | **Janssen** | 362 | 0.57 | 20.52 | 2.58 | 315.13 |
|  | Moderna | 1179 | 0.36 | 6.07 | 1.80 | 275.43 |
|  | **Pfizer** | 1531 | 0.47 | 5.16 | 2.83 | 1008.7 |
| limb discomfort | Janssen | 467 | 0.73 | 26.47 | 1.80 | 154.98 |
|  | **Moderna** | 2380 | 0.72 | 12.25 | 2.17 | 929.03 |
|  | Pfizer | 2073 | 0.64 | 6.98 | 1.79 | 483.2 |
| oropharyngeal discomfort | Janssen | 75 | 0.12 | 4.25 | 1.09 | 0.49 |
|  | Moderna | 649 | 0.20 | 3.34 | 2.38 | 306.49 |
|  | **Pfizer** | 781 | 0.24 | 2.63 | 3.41 | 675.46 |
| throat irritation | Janssen | 336 | 0.53 | 19.05 | 0.98 | 0.17 |
|  | Moderna | 2251 | 0.68 | 11.59 | 1.38 | 167.26 |
|  | **Pfizer** | 3323 | 1.03 | 11.19 | 2.57 | 1854.5 |
| disorientation | **Janssen** | 308 | 0.48 | 17.46 | 2.08 | 157.98 |
|  | Moderna | 985 | 0.30 | 5.07 | 1.32 | 54.88 |
|  | Pfizer | 1058 | 0.33 | 3.56 | 1.49 | 120.71 |
| vertigo | Janssen | 637 | 1.00 | 36.11 | 1.45 | 85.38 |
|  | Moderna | 3225 | 0.98 | 16.60 | 1.58 | 476.00 |
|  | **Pfizer** | 3859 | 1.19 | 13.00 | 2.13 | 1450.80 |
| feeling abnormal | **Janssen** | 2474 | 3.88 | 140.25 | 2.11 | 1340.3 |
|  | Moderna | 10147 | 3.08 | 52.23 | 1.93 | 2984.4 |
|  | Pfizer | 10025 | 3.10 | 33.77 | 1.94 | 3009.2 |
| feeling of body temperature change | **Janssen** | 217 | 0.34 | 12.30 | 2.87 | 234.21 |
|  | Moderna | 603 | 0.18 | 3.10 | 1.63 | 100.22 |
|  | Pfizer | 592 | 0.18 | 1.99 | 1.63 | 97.65 |
| hypoesthesia oral | Janssen | 2619 | 4.11 | 148.47 | 1.86 | 986.88 |
|  | Moderna | 8248 | 2.51 | 42.46 | 1.13 | 89.88 |
|  | **Pfizer** | 2604 | 0.81 | 8.77 | 2.79 | 1679.20 |
| axillary pain | Janssen | 175 | 0.27 | 9.92 | 0.57 | 56.88 |
|  | Moderna | 2394 | 0.73 | 12.32 | 1.81 | 569.91 |
|  | **Pfizer** | 3002 | 0.93 | 10.11 | 2.68 | 1802.00 |
| breast pain | Janssen | 119 | 0.19 | 6.75 | 1.24 | 5.10 |
|  | Moderna | 705 | 0.21 | 3.63 | 1.59 | 106.12 |
|  | **Pfizer** | 1017 | 0.31 | 3.43 | 2.95 | 716.01 |
| chest pain | Janssen | 2093 | 3.28 | 118.65 | 1.73 | 603.46 |
|  | Moderna | 7633 | 2.32 | 39.29 | 1.25 | 279.98 |
|  | **Pfizer** | 11968 | 3.70 | 40.31 | 2.52 | 6524.10 |
| eye pain | **Janssen** | 425 | 0.67 | 24.09 | 2.11 | 227.26 |
|  | Moderna | 1221 | 0.37 | 6.29 | 1.16 | 20.40 |
|  | Pfizer | 1472 | 0.46 | 4.96 | 1.54 | 193.95 |
| headache | **Janssen** | 15890 | 24.93 | 900.78 | 2.24 | 11188.00 |
|  | Moderna | 55574 | 16.89 | 286.08 | 1.65 | 10864 |
|  | Pfizer | 53483 | 16.55 | 180.16 | 1.59 | 9258.50 |
| migraine | **Janssen** | 1239 | 1.94 | 70.24 | 2.71 | 1198.10 |
|  | Moderna | 3598 | 1.09 | 18.52 | 1.61 | 576.85 |
|  | **Pfizer** | 4132 | 1.28 | 13.92 | 2.04 | 1396.60 |
| lymph node pain | Janssen | 150 | 0.24 | 8.50 | 0.58 | 45.45 |
|  | Moderna | 2048 | 0.62 | 10.54 | 1.85 | 525.95 |
|  | **Pfizer** | 2702 | 0.84 | 9.10 | 3.02 | 1977.50 |
| oropharyngeal pain | Janssen | 890 | 1.40 | 50.45 | 1.26 | 46.19 |
|  | Moderna | 4466 | 1.36 | 22.99 | 1.29 | 217.14 |
|  | **Pfizer** | 6060 | 1.88 | 20.41 | 2.07 | 2141.30 |
| parosmia | **Janssen** | 138 | 0.22 | 7.82 | 2.25 | 87.37 |
|  | Moderna | 507 | 0.15 | 2.61 | 1.79 | 116.80 |
|  | **Pfizer** | 704 | 0.22 | 2.37 | 3.19 | 556.56 |
| sensory disturbance | **Janssen** | 224 | 0.35 | 12.70 | 2.60 | 196.94 |
|  | Moderna | 367 | 0.11 | 1.89 | 0.72 | 32.43 |
|  | Pfizer | 507 | 0.16 | 1.71 | 1.11 | 4.22 |
| throat tightness | Janssen | 369 | 0.58 | 20.92 | 0.89 | 4.66 |
|  | Moderna | 2466 | 0.75 | 12.69 | 1.22 | 70.87 |
|  | **Pfizer** | 3708 | 1.15 | 12.49 | 2.28 | 1625.5 |
| unconsciousness | **Janssen** | 1831 | 2.87 | 103.80 | 2.26 | 1177.80 |
|  | Moderna | 3790 | 1.15 | 19.51 | 0.82 | 116.74 |
|  | Pfizer | 5201 | 1.61 | 17.52 | 1.27 | 222.79 |
| unresponsive to stimuli | **Janssen** | 632 | 0.99 | 35.83 | 2.02 | 299.13 |
|  | Moderna | 1393 | 0.42 | 7.17 | 0.79 | 66.81 |
|  | Pfizer | 2404 | 0.74 | 8.10 | 1.66 | 433.54 |
| sleep disorder | **Janssen** | 1061 | 1.66 | 60.15 | 2.23 | 659.92 |
|  | Moderna | 3701 | 1.12 | 19.05 | 1.64 | 625.31 |
|  | Pfizer | 3324 | 1.03 | 11.20 | 1.43 | 313.25 |
| ***Cardiovascular AE*** (14) | | | | | | |
| hypertension | Janssen | 636 | 1.00 | 36.05 | 1.68 | 162.86 |
|  | Moderna | 2633 | 0.80 | 13.55 | 1.44 | 246.59 |
|  | **Pfizer** | 3327 | 1.03 | 11.21 | 2.49 | 1754.30 |
| atrial fibrillation | Janssen | 189 | 0.30 | 10.71 | 1.47 | 26.48 |
|  | **Moderna** | 1145 | 0.35 | 5.89 | 2.11 | 415.38 |
|  | **Pfizer** | 1251 | 0.39 | 4.21 | 2.50 | 660.61 |
| decreased heart rate | **Janssen** | 144 | 0.23 | 8.16 | 2.02 | 67.81 |
|  | Moderna | 393 | 0.12 | 2.02 | 1.03 | 0.23 |
|  | Pfizer | 591 | 0.18 | 1.99 | 1.86 | 154.57 |
| heart rate irregular | Janssen | 130 | 0.20 | 7.37 | 1.37 | 12.52 |
|  | Moderna | 698 | 0.21 | 3.59 | 1.60 | 106.16 |
|  | **Pfizer** | 943 | 0.29 | 3.18 | 2.63 | 548.00 |
| increased heart rate | Janssen | 1373 | 2.15 | 77.83 | 1.84 | 494.96 |
|  | Moderna | 5442 | 1.65 | 28.01 | 1.53 | 694.82 |
|  | **Pfizer** | 6946 | 2.15 | 23.40 | 2.27 | 3050.10 |
| palpitations | Janssen | 1151 | 1.81 | 65.25 | 1.58 | 230.29 |
|  | Moderna | 5631 | 1.71 | 28.99 | 1.68 | 1061.90 |
|  | **Pfizer** | 7385 | 2.29 | 24.88 | 2.67 | 4427.80 |
| myocardial infarction | **Janssen** | 162 | 0.25 | 9.18 | 2.11 | 86.12 |
|  | Moderna | 581 | 0.18 | 2.99 | 1.58 | 84.57 |
|  | **Pfizer** | 740 | 0.23 | 2.49 | 2.35 | 345.59 |
| cerebrovascular accident | **Janssen** | 439 | 0.69 | 24.89 | 2.94 | 498.29 |
|  | Moderna | 1256 | 0.38 | 6.47 | 1.76 | 274.76 |
|  | **Pfizer** | 1391 | 0.43 | 4.69 | 2.11 | 511.72 |
| epistaxis | **Janssen** | 309 | 0.48 | 17.52 | 2.17 | 178.72 |
|  | Moderna | 875 | 0.27 | 4.50 | 1.18 | 18.07 |
|  | Pfizer | 1202 | 0.37 | 4.05 | 1.90 | 335.75 |
| contusion | **Janssen** | 644 | 1.01 | 36.51 | 2.03 | 311.33 |
|  | Moderna | 1768 | 0.54 | 9.10 | 1.05 | 2.70 |
|  | Pfizer | 1620 | 0.50 | 5.46 | 0.96 | 2.57 |
| pulmonary embolism | **Janssen** | 584 | 0.92 | 33.11 | 4.57 | 1351.53 |
|  | Moderna | 1217 | 0.37 | 6.26 | 1.93 | 351.01 |
|  | **Pfizer** | 1339 | 0.41 | 4.51 | 2.30 | 599.16 |
| thrombosis | **Janssen** | 1004 | 1.58 | 56.92 | 6.66 | 3716.05 |
|  | Moderna | 1205 | 0.37 | 6.20 | 1.34 | 73.55 |
|  | **Pfizer** | 1737 | 0.54 | 5.85 | 2.38 | 830.80 |
| pulmonary thrombosis | **Janssen** | 139 | 0.22 | 7.88 | 5.76 | 433.54 |
|  | Moderna | 225 | 0.07 | 1.16 | 1.75 | 47.82 |
|  | Pfizer | 286 | 0.09 | 0.96 | 2.64 | 166.38 |
| deep vein thrombosis | **Janssen** | 524 | 0.82 | 29.70 | 5.85 | 1667.82 |
|  | Moderna | 831 | 0.25 | 4.28 | 1.73 | 169.78 |
|  | **Pfizer** | 943 | 0.29 | 3.18 | 2.15 | 361.52 |
| ***Ear AE*** (4) | | | | | | |
| deafness | Janssen | 115 | 0.18 | 6.52 | 1.20 | 3.75 |
|  | Moderna | 498 | 0.15 | 2.56 | 1.00 | 0.00 |
|  | **Pfizer** | 809 | 0.25 | 2.73 | 2.03 | 269.64 |
| ear discomfort | Janssen | 225 | 0.35 | 12.75 | 1.80 | 74.52 |
|  | Moderna | 982 | 0.30 | 5.06 | 1.71 | 192.73 |
|  | **Pfizer** | 1362 | 0.42 | 4.59 | 3.00 | 982.30 |
| hypoacusis | Janssen | 154 | 0.24 | 8.73 | 1.95 | 66.02 |
|  | Moderna | 486 | 0.15 | 2.50 | 1.20 | 11.86 |
|  | **Pfizer** | 781 | 0.24 | 2.63 | 2.50 | 411.39 |
| tinnitus | Janssen | 1272 | 2.00 | 72.11 | 1.99 | 580.76 |
|  | Moderna | 5146 | 1.56 | 26.49 | 1.75 | 1100.70 |
|  | **Pfizer** | 6933 | 2.15 | 23.35 | 2.92 | 4844.67 |
| ***Eye AE*** (2) | | | | | | |
| vision blurred | **Janssen** | 1136 | 1.78 | 64.40 | 2.81 | 1178.67 |
|  | Moderna | 2531 | 0.77 | 13.03 | 1.16 | 44.01 |
|  | Pfizer | 3331 | 1.03 | 11.22 | 1.76 | 727.79 |
| visual impairment | **Janssen** | 519 | 0.81 | 29.42 | 2.26 | 332.92 |
|  | Moderna | 91 | 0.03 | 0.47 | 0.76 | 5.63 |
|  | Pfizer | 98 | 0.03 | 0.33 | 0.85 | 2.01 |
| ***Female reproductive system AE***  (5) | | | | | | |
| menstrual disorder | **Janssen** | 231 | 0.36 | 13.09 | 2.10 | 121.37 |
|  | Moderna | 773 | 0.23 | 3.98 | 1.42 | 68.29 |
|  | **Pfizer** | 1436 | 0.44 | 4.84 | 4.18 | 1592.20 |
| dysmenorrhoea | **Janssen** | 173 | 0.27 | 9.81 | 2.08 | 89.31 |
|  | Moderna | 568 | 0.17 | 2.92 | 1.37 | 41.04 |
|  | **Pfizer** | 1090 | 0.34 | 3.67 | 4.25 | 1230.30 |
| heavy menstrual bleeding | **Janssen** | 444 | 0.70 | 25.17 | 2.49 | 355.35 |
|  | Moderna | 1349 | 0.41 | 6.94 | 1.54 | 177.89 |
|  | **Pfizer** | 2508 | 0.78 | 8.45 | 4.77 | 3183.13 |
| menstruation irregular | **Janssen** | 316 | 0.50 | 17.91 | 2.17 | 182.57 |
|  | Moderna | 998 | 0.30 | 5.14 | 1.37 | 71.62 |
|  | **Pfizer** | 1875 | 0.58 | 6.32 | 4.05 | 2009.78 |
| vaginal hemorrhage | Janssen | 156 | 0.24 | 8.84 | 1.95 | 66.70 |
|  | Moderna | 487 | 0.15 | 2.51 | 1.18 | 9.95 |
|  | **Pfizer** | 870 | 0.27 | 2.93 | 2.95 | 612.07 |
| ***Investigation result abnormal AE*** (1) | | | | | | |
| oxygen saturation decreased | Janssen | 191 | 0.30 | 10.83 | 1.80 | 62.53 |
|  | Moderna | 731 | 0.22 | 3.76 | 1.41 | 60.71 |
|  | **Pfizer** | 942 | 0.29 | 3.17 | 2.10 | 340.84 |
| ***Homeostasis AE*** (5) | | | | | | |
| edema limbs | Janssen | 1269 | 1.99 | 71.94 | 1.14 | 22.06 |
|  | **Moderna** | 10143 | 3.08 | 52.21 | 2.26 | 4365.30 |
|  | Pfizer | 6036 | 1.87 | 20.33 | 1.09 | 32.15 |
| pharyngeal edema | Janssen | 292 | 0.46 | 16.55 | 1.47 | 41.34 |
|  | **Moderna** | 1810 | 0.55 | 9.32 | 2.20 | 722.26 |
|  | **Pfizer** | 2313 | 0.72 | 7.79 | 3.49 | 2071.88 |
| tongue edema | Janssen | 242 | 0.38 | 13.72 | 0.92 | 1.59 |
|  | Moderna | 1910 | 0.58 | 9.83 | 1.60 | 298.16 |
|  | **Pfizer** | 2421 | 0.75 | 8.16 | 2.38 | 1159.44 |
| thirst | **Janssen** | 200 | 0.31 | 11.34 | 2.33 | 136.87 |
|  | Moderna | 591 | 0.18 | 3.04 | 1.36 | 40.58 |
|  | Pfizer | 632 | 0.20 | 2.13 | 1.53 | 81.33 |
| hypoxia | **Janssen** | 291 | 0.46 | 16.50 | 2.12 | 157.46 |
|  | Moderna | 802 | 0.24 | 4.13 | 1.10 | 6.04 |
|  | Pfizer | 1042 | 0.32 | 3.51 | 1.63 | 171.83 |
| ***Immune system AE*** (4) | | | | | | |
| COVID-19 pneumonia | **Janssen** | 385 | 0.60 | 21.82 | 3.75 | 664.63 |
|  | Moderna | 826 | 0.25 | 4.25 | 1.57 | 117.90 |
|  | **Pfizer** | 1412 | 0.44 | 4.76 | 4.06 | 1517.44 |
| myocarditis | Janssen | 73 | 0.11 | 4.14 | 0.57 | 22.76 |
|  | Moderna | 783 | 0.24 | 4.03 | 1.29 | 37.54 |
|  | **Pfizer** | 1542 | 0.48 | 5.19 | 4.09 | 1669.49 |
| pericarditis | Janssen | 94 | 0.15 | 5.33 | 1.00 | 0.00 |
|  | Moderna | 582 | 0.18 | 3.00 | 1.28 | 25.79 |
|  | **Pfizer** | 975 | 0.30 | 3.28 | 2.93 | 676.20 |
| lymphadenopathy | Janssen | 642 | 1.01 | 36.39 | 0.48 | 361.76 |
|  | Moderna | 8877 | 2.70 | 45.70 | 1.48 | 882.18 |
|  | **Pfizer** | 11810 | 3.65 | 39.78 | 2.29 | 5303.36 |
| ***Injury AE*** (1) | | | | | | |
| head injury | **Janssen** | 310 | 0.01 | 17.57 | 2.07 | 156.62 |
|  | Moderna | 693 | 0.35 | 3.57 | 0.82 | 21.84 |
|  | Pfizer | 976 | 0.06 | 3.29 | 1.31 | 52.72 |
| ***Local AE***  (9) | | | | | | |
| injection-site erythema | Janssen | 7 | 0.01 | 0.40 | 0.02 | 324.32 |
|  | **Moderna** | 5670 | 1.72 | 29.19 | 11.31 | 12500.15 |
|  | Pfizer | 1355 | 0.42 | 4.56 | 0.79 | 61.86 |
| injection-site induration | Janssen | 2 | 0.00 | 0.11 | 0.03 | 68.95 |
|  | **Moderna** | 1205 | 0.37 | 6.20 | 10.47 | 2557.19 |
|  | Pfizer | 104 | 0.03 | 0.35 | 0.24 | 226.87 |
| injection-site mass | Janssen | 6 | 0.01 | 0.34 | 0.09 | 52.22 |
|  | **Moderna** | 838 | 0.25 | 4.31 | 5.08 | 1109.98 |
|  | Pfizer | 500 | 0.15 | 1.68 | 1.94 | 147.90 |
| injection-site pain | Janssen | 85 | 0.13 | 4.82 | 0.11 | 596.21 |
|  | **Moderna** | 9006 | 2.74 | 46.36 | 4.08 | 9701.66 |
|  | **Pfizer** | 6436 | 1.99 | 21.68 | 2.23 | 2708.10 |
| injection-site pruritus | Janssen | 266 | 0.42 | 15.08 | 0.17 | 1097.23 |
|  | **Moderna** | 20473 | 6.22 | 105.39 | 4.88 | 26765.79 |
|  | Pfizer | 2893 | 0.90 | 9.74 | 0.32 | 3920.40 |
| injection-site rash | Janssen | 153 | 0.24 | 8.67 | 0.15 | 748.77 |
|  | **Moderna** | 11805 | 3.59 | 60.77 | 3.71 | 11407.69 |
|  | Pfizer | 1660 | 0.51 | 5.59 | 0.28 | 2926.82 |
| injection-site reaction | Janssen | 9 | 0.01 | 0.51 | 0.12 | 56.30 |
|  | **Moderna** | 1160 | 0.35 | 5.97 | 8.68 | 2240.94 |
|  | Pfizer | 207 | 0.06 | 0.70 | 0.52 | 82 |
| injection-site swelling | Janssen | 15 | 0.02 | 0.85 | 0.05 | 289.48 |
|  | **Moderna** | 4312 | 1.31 | 22.20 | 5.54 | 6180.23 |
|  | Pfizer | 1501 | 0.46 | 5.06 | 0.96 | 1.65 |
| injection-site warmth | Janssen | 2 | 0.00 | 0.11 | 0.01 | 188.47 |
|  | **Moderna** | 3011 | 0.92 | 15.50 | 8.62 | 5805.98 |
|  | Pfizer | 578 | 0.18 | 1.95 | 0.56 | 172.69 |
| ***Musculoskeletal or connective tissue AE*** (2) | | | | | | |
| muscle spasm | **Janssen** | 1298 | 2.04 | 73.58 | 2.62 | 1168.26 |
|  | Moderna | 3126 | 0.95 | 16.09 | 1.19 | 70.33 |
|  | Pfizer | 3714 | 1.15 | 12.51 | 1.55 | 502.07 |
| muscle tightness | **Janssen** | 272 | 0.43 | 15.42 | 2.27 | 176.34 |
|  | Moderna | 835 | 0.25 | 4.30 | 1.40 | 66.68 |
|  | **Pfizer** | 1050 | 0.32 | 3.54 | 2.00 | 337.76 |
| ***Nervous system AE*** (1) | | | | | | |
| paresthesia oral | Janssen | 336 | 0.53 | 19.05 | 0.97 | 0.39 |
|  | Moderna | 2679 | 0.81 | 13.79 | 1.74 | 567.42 |
|  | **Pfizer** | 3627 | 1.12 | 12.22 | 2.94 | 2550.65 |
| ***Respiratory system AE*** (4) | | | | | | |
| acute respiratory failure | **Janssen** | 204 | 0.32 | 11.56 | 3.11 | 255.31 |
|  | Moderna | 515 | 0.16 | 2.65 | 1.57 | 73.05 |
|  | **Pfizer** | 840 | 0.26 | 2.83 | 3.68 | 801.76 |
| dyspnoea exertional | **Janssen** | 173 | 0.27 | 9.81 | 2.87 | 186.62 |
|  | Moderna | 459 | 0.14 | 2.36 | 1.53 | 57.57 |
|  | Pfizer | 661 | 0.20 | 2.23 | 2.79 | 424.63 |
| sinus congestion | **Janssen** | 133 | 0.21 | 7.54 | 2.11 | 70.86 |
|  | Moderna | 415 | 0.13 | 2.14 | 1.30 | 20.58 |
|  | Pfizer | 518 | 0.16 | 1.74 | 1.83 | 128.17 |
| respiratory tract congestion | Janssen | 278 | 0.44 | 15.76 | 1.35 | 23.27 |
|  | Moderna | 1156 | 0.35 | 5.95 | 1.09 | 6.37 |
|  | **Pfizer** | 1916 | 0.59 | 6.45 | 2.33 | 879.76 |
| ***Serious adverse event***  (1) | | | | | | |
| death | **Janssen** | 919 | 1.44 | 52.10 | 2.02 | 439.26 |
|  | Moderna | 3323 | 1.01 | 17.11 | 1.51 | 406.12 |
|  | Pfizer | 3587 | 1.11 | 12.08 | 1.73 | 751.74 |
| ***Skin AE*** (4) | | | | | | |
| flushing | Janssen | 665 | 1.04 | 37.70 | 1.25 | 32.74 |
|  | Moderna | 3469 | 1.05 | 17.86 | 1.35 | 231.79 |
|  | **Pfizer** | 4879 | 1.51 | 16.43 | 2.32 | 2225.60 |
| hot flushes | **Janssen** | 462 | 0.72 | 26.19 | 2.38 | 335.43 |
|  | Moderna | 1542 | 0.47 | 7.94 | 1.67 | 281.78 |
|  | **Pfizer** | 1792 | 0.55 | 6.04 | 2.16 | 694.20 |
| hyperhidrosis | **Janssen** | 3612 | 5.67 | 204.76 | 2.71 | 3554.09 |
|  | Moderna | 8891 | 2.70 | 45.77 | 1.28 | 403.28 |
|  | Pfizer | 10176 | 3.15 | 34.28 | 1.58 | 1542.20 |
| rash pruritic | Janssen | 448 | 0.70 | 25.40 | 0.72 | 49.51 |
|  | **Moderna** | 5336 | 1.62 | 27.47 | 2.08 | 1877.20 |
|  | Pfizer | 3046 | 0.94 | 10.26 | 0.96 | 3.26 |
| ***Urinary system AE*** (1) | | | | | | |
| acute kidney injury | **Janssen** | 137 | 0.21 | 7.77 | 2.36 | 97.44 |
|  | Moderna | 474 | 0.14 | 2.44 | 1.75 | 100.52 |
|  | **Pfizer** | 744 | 0.23 | 2.51 | 3.95 | 772.94 |
